# Impact of comorbidities and organ damage on hospital admissions and mortality in patients with sarcoidosis: an observational study from the Spanish National Registry

**DOI:** 10.1007/s11739-025-04221-w

**Published:** 2026-01-07

**Authors:** Susana Mellor-Pita, Víctor Moreno-Torres, Jorge Esteban-Sampedro, Mario Martín-Portugués, María Martínez-Urbistondo, Pablo Tutor-Ureta, Pedro Durán-del Campo, Román Fernández-Guitián, Rosalía Laporta-Hernández, Begoña Rodríguez, Raquel Castejón

**Affiliations:** 1 Internal Medicine Department, Health Research Institute Puerta de Hierro-Segovia de Arana (IDIPHIM) Hospital Universitario Puerta de Hierro Majadahonda, Madrid, Spain; 2 Medicine Department, School of Medicine, Universidad Autónoma de Madrid, Madrid, Spain; 3 UNIR Health Sciences School and Medical Center, Madrid, Spain; 4 Pneumology Depatrment, Health Research Institute Puerta de Hierro-Segovia de Arana (IDIPHIM) Hospital Universitario Puerta de Hierro Majadahonda, Madrid, Spain; 5 Nuclear Medicine Department, Health Research Institute Puerta de Hierro-Segovia de Arana (IDIPHIM) Hospital Universitario Puerta de Hierro Majadahonda, Madrid, Spain

**Keywords:** Sarcoidosis, Comorbidities, Mortality, Infection, Malignancies, Cardiovascular disease

## Abstract

Advanced lung disease, cardiovascular disease (CVD), infections, malignancies, and thromboembolic disease (TED) determine the mortality of patients with sarcoidosis. Our objective was to evaluate the main causes of admission and in-hospital mortality in patients with sarcoidosis in Spain. A retrospective and observational analysis of the National Registry of Hospital Discharges (RAE-CMBD) of patients admitted with a diagnosis of sarcoidosis between 2016 and 2021 was performed. A total of 18,887 admissions of patients with sarcoidosis were identified. The main causes of admission were infection (21%), sarcoidosis itself (16.4%), CVD (12.7%), and malignancies (7.1%). Overall, 892 (4.7%) patients died, mainly from infection (32.1%), CVD (16.3%) and neoplasms (12.4%), with case fatality rates of 7.2%, 6% and 8.3%, respectively. Factors associated with higher in-hospital mortality were Charlson comorbidity index (OR 1.30 95%CI 1.27–1.34), sarcoidosis pulmonary involvement (OR = 1.20, 95%CI 1.04–1.38), pulmonary fibrosis (OR 2.07; 95%CI 1.52–2.81) and CVD (OR 1.56; 95%CI 1.26–1.95), infection (OR 2.37; 95%CI 1.97–2.84), malignancies (OR 1.77; 95%CI 1.38–2.27) and TE-related admissions (OR 2.1, 95%CI 1.21–3.76). The main determinants of hospital admissions and mortality in patients with sarcoidosis are infections, CVD, neoplasm, VTE, pulmonary fibrosis, and a high comorbidity burden. While sarcoidosis itself is a common cause of admission, it is rarely the cause of death. Prevention of infections, VTE, and neoplasm, along with control of cardiovascular risk factors, may help reduce mortality in these patients.

## Introduction

Sarcoidosis is a chronic systemic autoimmune disease of unknown etiology characterized by the formation of non-caseating granulomas, primarily affecting the lungs, lymph nodes, skin, and eyes [1]. The prognosis of sarcoidosis is highly variable, ranging from spontaneous resolution to chronic inflammation complicated by fibrosis and/or organ damage, which occurs in 10–30% of cases [2]. This variability explains why mortality in patients with sarcoidosis is higher than in the general population, mainly due to advanced pulmonary disease with chronic respiratory failure, followed by cardiac and central nervous system (CNS) involvement [2, 3]. In addition, recent studies have highlighted an increased risk of cardiovascular disease (CVD), infections, neoplasms, and venous thromboembolism (VTE) as major contributors to premature death in this population [4–7].

Based on these considerations, the aim of our study is to evaluate the main causes of hospital admission and in-hospital mortality in patients with sarcoidosis in Spain, as well as to identify demographic and clinical factors associated with mortality in this population.

## Materials and methods

A retrospective and observational analysis was conducted using data extracted from the Spanish National Hospital Discharge Database (SNHDD), a publicly accessible registry managed by the Spanish Ministry of Health. The SNHDD includes demographic and epidemiological information and up to 20 discharge diagnoses per admission, coded since January 1, 2016, using the International Classification of Diseases, 10th Revision (ICD-10).

### Study population

We included all patients admitted between 2016 and 2021 with a diagnosis of sarcoidosis (code D86) in any diagnostic position. According to ICD-10 criteria, this encompasses patients with pulmonary, lymphatic, cutaneous, central nervous system (CNS), ocular, renal, cardiac, articular, and muscular involvement, as well as other manifestations such as uveoparotid fever or hepatic sarcoidosis, whether isolated or combined.

Demographic data and in-hospital outcomes such as age, sex, ethnicity, length of stay, admission to the intensive care unit (ICU), and death were obtained from the database. Baseline comorbidities were identified through additional ICD-10 codes recorded in the dataset, regardless of their position in each hospital admission episode. Based on this information, the Charlson Comorbidity Index (CCI) [8], was calculated, a well-validated scoring system that predicts clinical outcomes across a variety of diseases. It includes, among other conditions, diabetes mellitus, heart failure, dementia, chronic kidney disease, liver disease, and cancer.

Based on the structure and design of the database, the primary diagnosis was considered the main reason for admission and, when death occurred during hospitalization, the primary diagnosis of that episode was used as the cause of death. Therefore, all primary diagnoses were decoded and classified according to ICD-10, including sarcoidosis, CVD, infections, neoplasms, venous thromboembolism (VTE), among others. These diagnoses reflect those made by the attending physician during the hospital stay and subsequently coded using ICD-10.

Due to the design of the SNHDD, potential biases related to case identification and coding could not be fully controlled; however, focusing on categorical variables helps minimize misclassification.

### Statistical analysis

A descriptive analysis was performed to examine the causes of hospital admission and in-hospital mortality among patients with sarcoidosis hospitalized in Spain between 2016 and 2021. Categorical variables were reported as frequencies and percentages, while continuous variables were presented as means and standard deviations. Differences between groups were assessed using the Chi-square test or Student’s t-test, as appropriate.

In addition, binary logistic regression analysis was conducted to identify factors associated with mortality in patients hospitalized with sarcoidosis. The model was adjusted for sex, ethnicity, CCI, organ involvement due to sarcoidosis, and the most clinically relevant and fatal causes of admission, including sarcoidosis itself, CVD, infections, neoplasms, and VTE. A separate multivariable analysis was also performed to identify predictors of mortality specifically attributable to sarcoidosis. The model included sex, ethnicity, CCI, and the various organ involvements related to sarcoidosis.

A significance level of 0.05 was established for all analyses. Statistical analyses were performed using SPSS software, version 26.0 (IBM, Spain).

### Ethics

The study complies with the Declaration of Helsinki and was approved by the Ethics Committee of Hospital Universitario Puerta de Hierro Majadahonda-Madrid (Protocol number PI 162/24). All data were provided anonymously after the removal of all possible patient identifiers. Due to the design of the study and in accordance with Spanish legislation, informed consent was not required.

## Results

Between 2016 and 2021, a total of 18,887 hospital admissions of patients with sarcoidosis were identified in the SNHDD (Table 1). Of these, 53.1% were women, with a mean age of 62 years. A total of 76.6% were of Caucasian ethnicity. The distribution of organ involvement was as follows: pulmonary in 8,485 patients (45%), fibrosing pulmonary disease in 461 (2.4%), lymphatic in 2,565 (13.6%), cutaneous in 876 (4.61%), CNS in 118 (0.8%), ocular in 85 (0.5%), renal in 183 (1%), cardiac in 246 (1.3%), articular in 186 (1%), myositis in 37 (0.2%), and other forms in 1,906 (10.1%). The most frequent comorbidities were diabetes mellitus (27.6%), chronic pulmonary disease (23.8%), heart failure (23.1%), and chronic kidney disease (14.7%). Based on these data, the mean CCI was 4.9. Regarding outcomes, the mean hospital stay was 8.6 days, the ICU admission rate was 5.9%, and overall in-hospital mortality was 4.7%.

**Table 1:** Patient’s characteristics

Patients admitted with sarcoidosis (*N* = 18,887)	
Patient characteristics	
Women, *N* (%)	10,026 (53.1)
Age (years) (mean, SD)	62 (15.6)
Race *N* (%)	
Arab	450 (2.4)
Asian	29 (0.2)
African American	276 (1.5)
Caucasian	14,445 (76.6)
Indian	22 (0.1)
Latin/Hispanic	237 (1.3)
Sarcoidosis organ involvement *N* (%)	
Lung	8485 (45)
Fibrosing pulmonary disease	461 (2.4)
Lymph node	2565 (13.6)
Skin	876 (4.6)
Central nervous system	118 (0.8)
Ocular	85 (0.5)
Kidney	183 (1)
Cardiac	246 (1.3)
Articular	186 (1)
Myositis	37 (0.2)
Other	1906 (10.1)
Comorbidities *N* (%)	
Peripheral vascular disease	1859 (9.9)
Ischemic heart disease	590 (3.1)
Heart failure	4354 (23.1)
Cerebrovascular disease	330 (1.7)
Dementia	590 (3.1)
Diabetes	5198 (27.6)
Chronic pulmonary disease	4486 (23.8)
Liver disease	1676 (8.9)
Peptic ulcer disease	130 (0.7)
Chronic kidney disease	2778 (14.7)
Leukemia	174 (0.9)
Lymphoma	490 (2.6)
Myelodysplastic syndrome	95 (0.5)
Solid organ neoplasm	925 (4.9)
Metastatic solid organ neoplasm	667 (3.5)
AIDS	45 (0.2)
Charlson comorbidity index (mean, SD)	4.9 (2.8)
Outcomes	
Death, *N* (%)	892 (4.7)
Average admission length (days), (mean, SD)	8.6 (11.2)
ICU admission, *N* (%)	1117 (5.9)
ICU admission length (days), (mean, SD)	7.1 (15.1)

*SD* Standard deviation, *ICU* Intensive care unit, *AIDS* Acquired immunodeficiency syndrome

### Causes of admission and in-hospital mortality

Table 2 describes the causes of admission and in-hospital mortality among patients hospitalized with sarcoidosis, as well as the case fatality rate for each diagnostic category. During the study period, the most frequent causes of admission were infections (21%), followed by sarcoidosis itself (16.4%), CVD (12.7%), and neoplasms (7.1%). Patients admitted due to sarcoidosis were younger (54 vs. 64.7 years, p < 0.001) and had a lower CCI score (3.2 vs. 5.2, p < 0.001) compared to those admitted for other causes.

**Table 2:** Causes of admission and death

	Admissions	Age (mean, SD)	Death (%)	Mortality rate (%)	Age (mean, SD)
	18,887	62 (15.6)	892	4.7	72.9 (12.6)
Infections	3961 (21)	66.2 (14.9)	286 (32.1)	7.2	74.3 (11.3)
Sarcoidosis	3102 (16.4)	54 (15.7)	68 (7.6)	2.2	66 (14.7)
Cardiovascular	2397 (12.7)	69.3 (13.1)	145 (16.3)	6	75.7 (12.2)
Neoplasm	1331 (7.1)	63.8 (12.3)	111 (12.4)	8.3	67.8 (12.9)
Respiratory disease	1738 (9.2)	66.6 (14.1)	108 (12.1)	6.2	74 (11.5)
Digestive-surgery	1248 (6.6)	63.5 (14.2)	43 (4.8)	3.4	74.1 (11.9)
Osteo-articular	588 (3.1)	62.9 (13.8)	7 (0.8)	1.2	81.7 (7.1)
Traumatic	534 (2.8)	69.5 (14.7)	23 (2.6)	4.3	75.1 (15.9)
Urological	297 (1.6)	58.8 (13.8)	2 (0.2)	0.7	83.5 (14.8)
Neurological	296 (1.6)	60.3 (15.4)	14 (1.6)	4.7	69.6 (12.2)
Hematologic	269 (1.4)	61.2 (17.1)	11 (1.2)	4.1	71.5 (10.2)
Kidney disease	230 (1.2)	64.3 (13.9)	11 (1.2)	4.8	75.3 (12.5)
Endocrine	224 (1.2)	62.1 (13.4)	3 (0.3)	1.3	67.3 (10.4)
VTE	213 (1.1)	68.2 (14.5)	14 (1.6)	6.6	76.1 (11.8)
Other	3693 (19.6)	60.2 (15.8)	89 (10)	2.4	72.3 (13)

*SD* Standard deviation, *VTE* Venous thromboembolism

A total of 892 patients (4.7%) with sarcoidosis died during hospitalization. Of those who died, 52.1% were women, with a mean age of 72.9 years. The leading causes of death were infections (32.1%), CVD (16.3%), and neoplasms (12.4%), with case fatality rates of 7.2%, 6%, and 8.3%, respectively. Notably, although VTE accounted for only 1.6% of deaths, it had a high case fatality rate of 6.6% due to its low prevalence (1.1%).

**Fig. 1:**
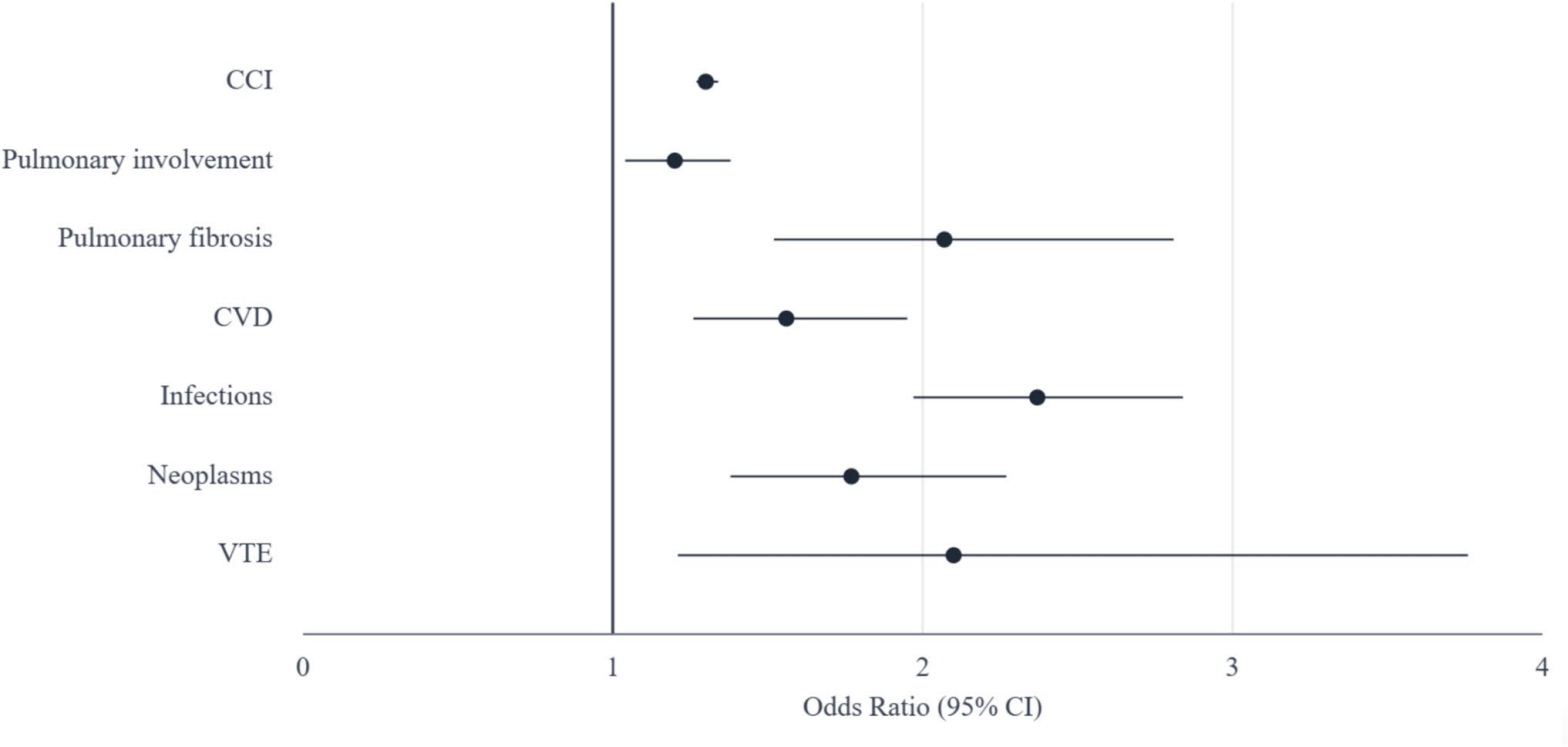
Factors associated with in-hospital mortality in patients admitted with sarcoidosis in Spain between 2016 and 2021. Forest plot representing the multivariate analysis by binary logistic regression of the factors associated with in-hospital mortality in patients admitted with sarcoidosis in Spain between 2016 and 2021. The model includes sex, ethnicity, the CCI, organ involvement by sarcoidosis, and the most clinically relevant and lethal reasons for admission, including sarcoidosis activity itself, CVD, infections, neoplasms, and VTE. The circles represent the Odds Ratio, and the lines represent the 95% confidence intervals. *CCI* Charlson Comorbidity Index, *CVD* Cardiovascular Disease, *VTE* Venous Thromboembolism

Multivariable analysis of in-hospital mortality showed that the factors independently associated with increased mortality were higher CCI (OR 1.30, 95% CI 1.27–1.34), pulmonary involvement (OR 1.20, 95% CI 1.04–1.38), presence of pulmonary fibrosis (OR 2.07, 95% CI 1.52–2.81), and admissions due to CVD (OR 1.56, 95% CI 1.26–1.95), infections (OR 2.37, 95% CI 1.97–2.84), neoplasms (OR 1.77, 95% CI 1.38–2.27), and VTE (OR 2.1, 95% CI 1.21–3.76) (Fig. 1).

**Fig. 2:**
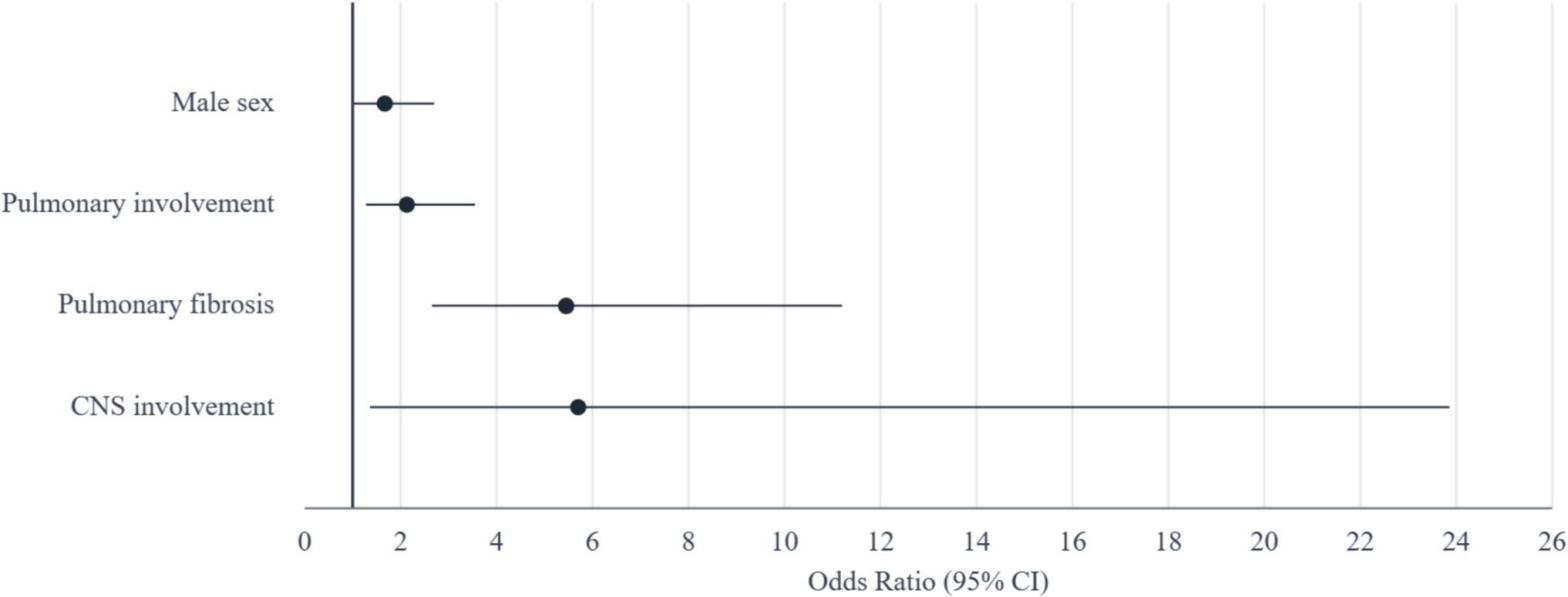
Factors associated with in-hospital mortality due to sarcoidosis in patients admitted with sarcoidosis in Spain between 2016 and 2021. Forest plot representing the multivariate analysis by binary logistic regression of factors associated with in-hospital mortality from sarcoidosis in patients admitted with sarcoidosis in Spain between 2016 and 2022. The model includes sex, ethnicity, the CCI, and organ involvement by sarcoidosis. The circles represent the Odds Ratio, and the lines represent the 95% confidence intervals. *CNS* Central Nervous System

Finally, sarcoidosis itself was the cause of in-hospital death in 68 patients (7.6%), with a lower case fatality rate compared to other conditions (2.2% vs. 5.2%, p < 0.001). Patients who died due to sarcoidosis were younger (66 vs. 73.4 years, p < 0.001), more likely to be male (60.3% vs. 46.8%, p = 0.022), and had a lower CCI (5.4 vs. 7.4, p < 0.001) compared to those who died from other causes. After adjustment, only male sex (OR 1.67, 95% CI 1.02–2.70), pulmonary involvement (OR 2.13, 95% CI 1.28–3.55), pulmonary fibrosis (OR 5.45, 95% CI 2.65–11.2), and CNS involvement (OR 5.7, 95% CI 1.36–23.86) were independently associated with in-hospital mortality due to sarcoidosis (Fig. 2).

## Discussion

This large nationwide epidemiological study details the causes of hospital admission and in-hospital mortality, as well as the factors that contribute to the increased mortality observed in patients with sarcoidosis compared to the general population [2, 3]. Recent studies have reported a rise in hospital admissions among patients with sarcoidosis [9]. However, there are few publications that analyze the main reasons for admission; as in our study, Gerke *et al*. and Ungprasert *et al*. found that comorbidities, rather than sarcoidosis itself, were the primary reason for hospitalization [10, 11]. Regarding mortality, infections were the leading cause of in-hospital death in our cohort, in contrast to previous studies in which sarcoidosis itself was reported as the main cause [12, 13]. Nevertheless, a growing body of evidence has recently identified infections, CVD, neoplasms, and VTE as major causes of hospital admission and death in this population, consistent with our findings [5, 14].

Over the past few years, it has become evident that patients with sarcoidosis have a 1.8- to twofold higher risk of severe infections compared to the general population, a risk that is even greater in those receiving immunosuppressive therapy [11, 15]. It is well known that glucocorticoids play a significant role in increasing susceptibility to infections. However, the sarcoid inflammatory process itself may also contribute, as patients with sarcoidosis not receiving active treatment still have a higher risk of infections compared to healthy individuals [11]. The underlying pathophysiological mechanism is not fully understood, but it is likely that inflammation and granuloma formation trigger a state of anergy to pathogens, which is further amplified by immunosuppressive therapy [16]. Based on this, it is essential to implement strategies to reduce infection risk in these patients, including the rational use of immunosuppressive drugs and glucocorticoids, up-to-date vaccination schedules, and prophylactic treatment for opportunistic infections when indicated [17].

In our study, CVD was the second leading cause of in-hospital mortality among patients with sarcoidosis, consistent with previous reports by other authors [12, 13]. There is evidence supporting an increased risk of CVD and related mortality in these patients [4], either as a consequence of direct cardiac involvement (heart failure, arrhythmias, cardiomyopathy), or due to accelerated atherosclerosis secondary to chronic systemic inflammation and prolonged steroid use, leading to higher prevalence and mortality from ischemic heart disease and cerebrovascular disease [18, 19]. This phenomenon has already been described in other inflammatory or autoimmune diseases, and in sarcoidosis, increased subclinical atherosclerosis has also been reported, raising the question of whether sarcoidosis should be considered an independent cardiovascular risk factor [20].

Third, neoplasms were another major cause and factor associated with in-hospital mortality in our cohort. Several studies have reported an increased risk of hematologic malignancies, primarily non-Hodgkin lymphoma, and, to a lesser extent, solid organ cancers (skin, upper gastrointestinal tract, kidney, liver, and colorectal) in association with sarcoidosis [21–23]. Similarly, patients with sarcoidosis have higher overall cancer mortality, particularly from hematologic malignancies, compared to the general population [6, 23, 24]. Some risk factors for carcinogenesis in sarcoidosis have been proposed, including chronic inflammation and impaired immune surveillance due to both the disease itself and immunosuppressive treatment [25, 26].

On the other hand, our epidemiological analysis highlights that although the prevalence of VTE during hospitalization was low (1.1%), its case fatality rate was high (6.6%). While some studies have demonstrated an association between sarcoidosis and VTE, findings regarding mortality remain controversial [14, 27]. For instance, Swigris *et al*. showed that patients with sarcoidosis have higher VTE-related mortality than the general population [12]. Similarly, Pohle *et al*. identified VTE as a mortality-associated factor in hospitalized patients with sarcoidosis [7]. However, more recent studies have not confirmed this increased mortality [27]. In our study, VTE was independently associated with in-hospital mortality. These discrepancies may be explained by differences in study populations or data sources (outpatient vs. inpatient settings, or data obtained from death certificates versus hospital records). In any case, the elevated VTE risk in sarcoidosis is likely related to local and systemic inflammatory activity promoting a hypercoagulable state, endothelial damage caused by pro-inflammatory cytokines and oxidative stress, as well as other contributing factors, such as the presence of antiphospholipid antibodies, which have been reported in up to 38% of patients with sarcoidosis [28, 29].

Finally, our study highlights that the clinical and temporal profile of patients admitted or who die due to sarcoidosis differs from that of patients admitted or who die from other causes such as infections, CVD, neoplasms, or VTE. Specifically, patients admitted or deceased due to sarcoidosis were, on average, 10 and 7 years younger, respectively, had a lower comorbidity burden, and showed significantly lower in-hospital mortality or case fatality rates. In this group, mortality was associated only with male sex, pulmonary involvement, pulmonary fibrosis, and CNS involvement, factors classically linked to more severe disease [1, 30]. In contrast, infections, CVD, neoplasms, and VTE may be considered long-term complications of sarcoidosis, occurring when the disease is less active, which may help explain the increased morbidity and mortality observed in this population compared to the general population [2, 30]. Thus, the high comorbidity burden observed in patients with sarcoidosis, including heart failure, kidney disease, and diabetes, among others, reflected in a mean CCI close to 5, together with pulmonary involvement, pulmonary fibrosis, CVD, infections, neoplasms, and VTE, are the main factors associated with mortality in the multivariable analysis of the entire study population. As well as identifying the main causes of death, our study also assessed the factors independently associated with in-hospital mortality.

Our study has several limitations. First, we lack important information regarding the course of the disease, clinical characteristics, and histopathological data, or exposures (e.g., smoking status), as well as the use of immunosuppressive drugs, which could have allowed for more robust conclusions. In parallel, the database does not provide biochemical parameters, bone health outcomes, or information on exposure to corticosteroids or anti-osteoporotic therapies, thereby precluding an assessment of their impact on disease burden and other relevant comorbidities in this population. Second, a major pitfall is that the SNHDD is based on administrative coding, and therefore the accuracy of the diagnoses cannot be verified, as information on the clinical, radiological, or histopathological criteria used by physicians is not available. Third, this analysis was restricted to hospital admissions, introducing a potential selection bias. However, we primarily evaluated categorical variables, such as causes of admission and mortality, which are less prone to misclassification. Therefore, we believe that our work, based on an analysis of a national registry with a significant sample size over a prolonged study period, yields consistent and insightful results regarding morbidity and mortality in patients with sarcoidosis.

In conclusion, the main determinants of mortality in patients with sarcoidosis are infections, CVD, neoplasms, and VTE, along with the presence of pulmonary fibrosis and a high comorbidity burden. Greater knowledge of the disease, early diagnosis, and timely initiation of immunosuppressive treatment when indicated may explain the lower mortality directly related to sarcoidosis activity. However, chronic inflammation, increased comorbidities, and immunosuppressive therapy likely contribute to higher mortality from infections, CVD, and neoplasms. Based on this, preventive measures—such as up-to-date vaccination schedules, appropriate management and control of cardiovascular risk factors, prevention and monitoring of VTE, and cancer screening programs, may help reduce mortality in this population.
